# Acute Decompensated Heart Failure Due to Combined Ischemic Cardiomyopathy and Cardiac Amyloidosis

**DOI:** 10.7759/cureus.32281

**Published:** 2022-12-07

**Authors:** Arinze N Bosah, Amel Tobaa, Kushani Gajjar, Kartikeya Kashyap, Craig Alpert

**Affiliations:** 1 Internal Medicine, Allegheny Health Network, Pittsburgh, USA; 2 Cardiology, Allegheny Health Network, Pittsburgh, USA

**Keywords:** heart failure, hfref, coronary artery disease, cardiac amyloidosis, mixed cardiomyopathy

## Abstract

There are no established guidelines for the management of concurrent ischemic cardiomyopathy and cardiac amyloidosis due to the rarity of this phenomenon. We present the case of an African American woman in her 70s who was found to be in acute decompensated heart failure after she presented with progressive dyspnea. Initial workup revealed severe left ventricular systolic dysfunction with an ejection fraction of 20% and severe multivessel coronary artery disease, including severe left main disease. Multimodality imaging with cardiac MRI and technetium-99m pyrophosphate scintigraphy (PYP) during this hospital course revealed concurrent cardiac amyloidosis. Her systolic dysfunction was attributed to a combination of cardiac amyloidosis and ischemic cardiomyopathy. A multidisciplinary team comprised of interventional cardiology, cardiac surgery, and advanced heart failure amyloid specialists worked collaboratively to formulate an optimal treatment plan based on their collective clinical experiences and the limited literature, which ultimately resulted in a positive clinical outcome. Further investigation is needed to define treatment strategies specific to this patient population.

## Introduction

The identification and diagnosis of cardiac amyloidosis (CA) are increasing exponentially due to advances in multimodality cardiac imaging coupled with better disease awareness. Interestingly, among patients with CA, some have observed a lower prevalence of significant coronary artery calcification [1]. Perhaps this explains the absence of specific guidelines that address the management of mixed cardiomyopathy due to concomitant CA and significant coronary artery disease (CAD). We present a case of coexisting cardiac transthyretin amyloidosis (ATTR) and multi-vessel CAD manifesting as acute decompensated heart failure with reduced ejection fraction. 

## Case presentation

A 76-year-old African American woman with a history of hypertension, type 2 diabetes mellitus, chronic kidney disease stage 3, and longstanding bilateral carpal tunnel syndrome presented to an outside hospital with progressive dyspnea, paroxysmal nocturnal dyspnea, and orthopnea and was diagnosed with a new cardiomyopathy. She described worsening functional status, worsening dyspnea on exertion, and inability to climb a flight of stairs in the past month. She denied any chest pain, palpitations, lightheadedness, or syncope. She also endorsed lower leg edema above her knees as well as swelling in her abdomen. Her initial evaluation revealed severe triple-vessel coronary artery disease (CAD), including severe distal left main CAD (80%-90% calcified stenosis), Left anterior descending disease (80% ostial stenosis), left circumflex disease (proximal 75% stenosis before the bifurcation) and left ventricular ejection fraction (LVEF) of 20%, prompting transfer to our tertiary facility for consideration for revascularization via high-risk percutaneous coronary intervention (PCI) or coronary artery bypass surgery (CABG). 

**Figure 1:**
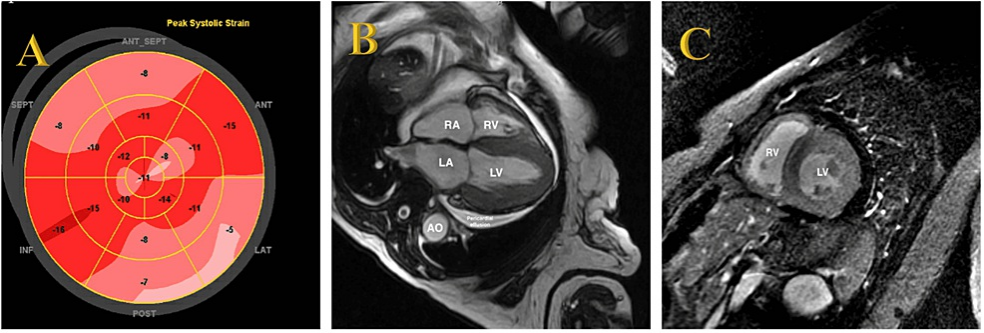
Transthoracic echocardiogram strain imaging and cardiac magnetic resonance image (CMRI). A. Apical sparing of longitudinal systolic strain (GLS -13.2%) consistent with cardiac amyloidosis. B. SSFP: Steady state free precession sequence demonstrating bi-atrial dilation and thickened left ventricular wall with moderate-sized pericardial effusion (LV: Left ventricle, RV: Right ventricle, LA: Left atrium, RA: Right atrium, Ao: Thoracic aorta). C. Look-Locker inversion recovery sequences with Late Gadolinium Enhancement (LGE) demonstrating diffuse dense circumferential and transmural left and right ventricular enhancement and Query Amyloid Late Enhancement (QALE) score of 18 most consistent with cardiac transthyretin amyloidosis [>13 scores, 82% sensitive, 76% specific for differentiating ATTR and AL]. (LV: Left ventricle, RV: Right ventricle).

On arrival, her vital signs were stable (temperature 36.9° C, blood pressure 108/54 mm Hg, heart rate 78 beats per minute, saturation 98% on two liters of supplemental oxygen via nasal cannula). Initial electrocardiogram showed normal sinus rhythm with a ventricular rate of 80, left axis deviation, and poor R wave progression in anterior leads but no evidence of low voltage QRS complexes. Her examination revealed diminished breath sounds without evidence of jugular venous distention or lower extremity edema. Her labs revealed creatinine 1.67 mg/dL (0.50-0.90 mg/dL), total protein 6.8 g/dL (6.2-8.1 g/dL), AST 56 U/L (0-32 U/L), ALT 44U/L ( 0-33 U/L), alkaline phosphatase 92 U/L (35-104 U/L), total bilirubin 0.7 mg/dL (0.0-1.7 mg/dL), troponin T 0.11 ng/mL (0.00 - 0.03 ng/mL), and Pro-BNP 5,560 pg/mL (0-299 pg/mL). A Swann- Ganz catheter was placed with initial numbers of central venous pressure (CVP) 8, pulmonary artery pressure (PAP) 42/17, cardiac output (CO) 2.87, cardiac index (CI) 1.5, and systemic vascular resistance (SVR) 2368. She was admitted to the cardiac intensive care unit for pulmonary artery catheter-guided optimization with diuretics and intravenous vasodilators. Surprisingly, a repeat transthoracic echocardiogram (TTE) with strain imaging revealed additional findings, including grade III diastolic dysfunction and an apical-sparing pattern (Figure 1A) suggestive of coexisting CA that was then corroborated by a cardiac magnetic resonance (CMR) demonstrating pericardial effusion, restrictive physiology, and characteristic late gadolinium enhancement. (Figures 1B, 1C). 

**Figure 2:**
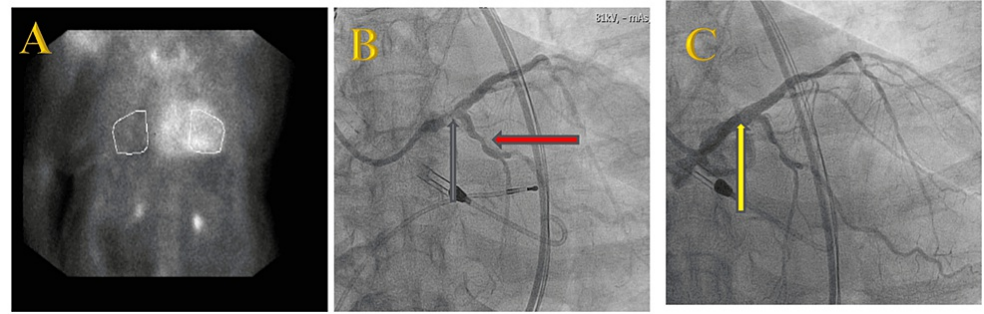
Technetium-99m pyrophosphate (PYP) scan and coronary angiogram A. Heart-to-contralateral (H/CL) ratio is 1.82 at one hour after tracer injection. H/CL ratios of >1.5 at one hour are strongly suggestive of ATTR, while ratios <1.5 are not consistent with ATTR. B. Eccentric 80-90% calcified stenosis of the distal left main (LM) artery extending into the ostial left anterior descending (LAD) artery (blue arrow) and residual stenosis involving the left circumflex artery (red arrow). C. Patent LM artery (yellow arrow) with TIMI 3 flow following atherectomy and stent placement.

Ultimately, after immunofixation electrophoresis proved to be normal [ Free kappa light chains 3.28 mg/dL(0.33-1.94 mg/dL), free lambda light chain 2.53 mg/dL (0.26-1.65 mg/dL), kappa/lambda light chain ratio 1.30 (0.26-1.65)], transthyretin cardiac amyloidosis was diagnosed with positive technetium-99m Pyrophosphate Scintigraphy (PYP) (Figure 2A) with grade 3 uptake using the Perugini staging system. Her systolic heart failure was therefore attributed to a combination of significant CAD and ATTR CA. The cardiac surgery team determined her perioperative risk for CABG to be prohibitive due to advanced age, ATTR, and low LVEF. Therefore, she instead underwent successful high-risk PCI to her unprotected left main artery extending into the left anterior descending artery using an Impella CP for temporary mechanical circulatory support (Figures 2B, 2C). She recovered well and was discharged on both dual-antiplatelet therapy and guideline-directed medical therapy (GDMT).

The patient was seen for follow-up at the Multidisciplinary CA Clinic and had genetic testing that revealed a pathogenic V122I mutation suggestive of hereditary ATTR. She underwent nerve conduction studies which did not demonstrate neuropathy and was subsequently started on tafamidis. The combination of effective revascularization, GDMT, and ATTR-directed treatment resulted in a favorable outcome. She had a significant clinical improvement 6 weeks from the index hospitalization from NYHA Class IV to NYHA II and LVEF improvement from 20% to 50%.

## Discussion

This case highlights several noteworthy features: a rare occurrence of combined ischemic and amyloid cardiomyopathy causing acute heart failure, a successful multidisciplinary approach to revascularization, and the importance of considering CA even when an alternative diagnosis has been established. 

CA is a well-established cause of heart failure with preserved ejection fraction. Greater than 95% of the cases of cardiac amyloidosis are caused by two predominant types: transthyretin amyloidosis (ATTR) and light chain amyloidosis (AL) [2]. Cardiac amyloidosis is usually suspected in patients with the typical presentation of heart failure along with low QRS voltage on ECG, echocardiographic findings of restrictive physiology, left ventricle (LV) wall thickness, biatrial enlargement, and pericardiac effusion [3,4]. A classical pattern of myocardial strain (apical sparing) may also be present in TTE. Next, the existence of plasma dyscrasia would need to be excluded with normal serum and urine electrophoresis with immunofixation and serum-free light chains [3,4]. The presence of plasma dyscrasia points towards a diagnosis of AL amyloidosis, which warrants a biopsy to confirm. Unlike AL amyloidosis, ATTR can be confirmed with a positive PYP scan without the need for a biopsy. Our patient was diagnosed with hereditary ATTR, which occurs when an autosomal-dominant mutation drives the production of unstable transthyretin protein [3]. This unstable protein is subsequently deposited in the myocardial tissue, leading to biventricular wall thickening and stiffening that compromise ventricular filling despite preserved systolic function. Over time, however, progressive ATTR can also cause heart failure with reduced ejection fraction (HFrEF), perhaps due to progressive microvascular and even macrovascular dysfunction [1,4-6]. Interestingly, CA mainly affects intramural coronary arteries, while true intraluminal epicardial coronary obstruction may occur less frequently in patients with CA [1]. By extension, there remains a paucity of literature demonstrating outcomes of surgical revascularization among CA patients. However, case reports suggest ample reason for pessimism and favor a percutaneous approach [7]. 

Not only is revascularization challenging in CA patients, but often, CA patients with HFrEF are also unable to tolerate meaningful GDMT due to worsening bradycardia and hypotension [6,7]. As a result, our patient required particularly close follow-up to ensure the safe optimization of GDMT, which included metoprolol succinate, sacubitril-valsartan, hydralazine, and isosorbide monohydrate.

Our case appears to be just the second reported case of concurrent amyloid and ischemic cardiomyopathy causing decompensated heart failure and required a coordinated multidisciplinary treatment approach given the lack of applicable guidelines. In this case, a multidisciplinary team comprised of CT surgery, interventional cardiology, and advanced heart failure amyloid specialist convened to formulate an optimal treatment plan, drawing upon the limited literature and collective clinical experience. 

## Conclusions

Cardiac amyloidosis is known to be underdiagnosed, but it is not as rare as once believed now that diagnosis no longer requires invasive endomyocardial biopsy. As CA is diagnosed with increasing frequency, we suspect that the prevalence of mixed cardiomyopathy will only continue to rise. Given the unique challenges in treating these patients, further investigation is needed to characterize treatment strategies specific to this population with concomitant ischemic and amyloid cardiomyopathy. For now, a multidisciplinary approach must be developed to individualize the care of such patients.
